# Genome size and identification of repetitive DNA sequences using low coverage sequencing in *Hancornia speciosa* Gomes (Apocynaceae: Gentianales)

**DOI:** 10.1590/1678-4685-GMB-2019-0175

**Published:** 2020-11-09

**Authors:** Vanessa Santos, Edson Ferreira da Silva, Cícero Almeida

**Affiliations:** ^1^ Universidade Federal Rural de Pernambuco, Recife, PE, Brazil.; ^2^ Universidade Federal de Alagoas, Laboratório de Recursos Genéticos, Arapiraca, AL, Brazil.

**Keywords:** NGS, mangaba, satDNA, chromovirus, evolution

## Abstract

Repetitive DNA is an important component of eukaryotic genomes, accounting for more than 90% of the genome size of some species, including mobile elements and satellite DNA sequences. The aim of study was to characterize the genome of *Hancornia speciosa* Gomes using C-value genome size estimate and repetitive DNA sequences analysis. The genome size estimate was obtained by flow cytometry and the repetitive DNA sequences were accessed using graph-based clustering. Evolutionary relationships among species of Apocynaceae was obtained using reads of *Catharanthus roseus* L., *Rhayza stricta* Decne, and *Asclepias syriaca* L. from the NCBI and analyzed by graph-based clustering. The genome size estimates in two botanical varieties showed 2C-values ranging from 0.88 to 1.08 pg, indicating small genome size. Clusters representing repeats making up at least 0.01% of the genome revealed the proportion of repetitive DNA ranging from 19.87% (*H. speciosa*) to 51.674% (*A. syriaca*), of which the mobile elements were more abundant. Satellite DNA sequences were not found in *H. speciosa* and *R. stricta*, while at least one satellite was detected in *C. roseus* and *A. syriaca*, suggesting that the LTR retrotransposon Ty3/Gypsy/Chromovirus may have replaced the satellite DNA in *H. speciosa* and *R. stricta*.

Repetitive DNA is the most relevant component in genome evolution and consists of several large classes, among which the transposable elements (TEs) and satellite DNA sequences (satDNA) are the most important (Biscotti *et al.*, 2015). TEs are generally the most abundant and can be classified into retrotransposons or Class I (“copy and paste”) and transposons or Class II (Wicker *et al.*, 2007; Rebollo *et al.*, 2012). The second most abundant class is satDNA, which is characterized by long arrays of tandemly arranged units (known as monomers). SatDNAs are the main components of constitutive heterochromatin, spanning up to several megabases in length with high evolutionary dynamics and high intra- and inter-specific sequence diversity, of which most satellite families are species- or genus-specific (Macas *et al.*, 2002; Belyayev *et al.*, 2019).[Bibr B1]

*Hancornia speciosa* Gomes (family Apocynaceae, Gentianales) is a fruit tree from tropical and subtropical regions. The species has a broad geographic distribution in Brazil, occurs in Caatinga, Cerrado and in two ecoregions of the Atlantic Rain Forest: the Coastal Tablelands and *Restinga* (Lima and Scariot, 2010). The species also occurs in Paraguay, Bolivia and Peru (Collevatti *et al.*, 2016). *H. speciosa* is an economically important plant, popularly known as “mangaba” or “mangabeira,” and is consumed as candy, ice-cream, juice or “*in natura*”, and the fruits have high concentration of vitamin C (Lima and Scariot, 2010; Santos *et al.*, 2017). The leaves have medicinal properties for treating diabetes (Pereira *et al.*, 2015) and blood pressure (Silva *et al.*, 2016). The latex has been reported to have medicinal properties to treat ulcers, gastritis and tuberculosis (Ribeiro *et al.*, 2016).[Bibr B22]

*Hancornia speciosa* is poor in molecular genetics studies and there is no information about the genome size, evolution or genome organization. In addition, monoculture of crop plants and urban expansion have reduced the natural populations. The Apocynaceae family has a robust phylogeny, containing monophyletic and paraphyletic subfamilies; however, some tribes and the paraphyletic Rauvolfioideae and Apocynoideae subfamilies require new studies for a better understanding of paraphyly (Fishbein *et al.*, 2018). *H. speciosa* belongs to the Rauvolfioideae subfamily, and genomic studies, including repetitive DNA contribute to understanding paraphyly in Rauvolfioideae and the relationships among Apocynaceae species. The aims of this study were to determine the genome size and characterize the repetitive DNA in *H. speciosa*. We also explored high-throughput sequencing to characterize the repetitive fractions for a better understanding of *H. speciosa* genomic evolution. The following questions were of interest: (1) What is the genome size estimate of *H. speciosa*? (2) What are the components and amounts of the repetitive fractions in the *H. speciosa* genome? (3) What are the genomic relationships of *S. speciosa* with to other species of Apocynaceae family.[Bibr B10]

Genome size was determined by flow cytometry. A suspension of nuclei from young leaves was prepared as described by Dolezel *et al.* (2007) using WPB buffer. The genome sizes were estimated using a CyFlow SL flow cytometer (Partec, Görlitz, Germany). Final DNA content was calculated for each accession based on at least three different measurements. The young leaves of *Solanum lycopersium* L. (1C = 1.96 pg DNA) (Dolezel *et al.*, 2010) were used as an internal control. FloMax software (Partec) was used for data processing. Genome size was estimated for two botanical varieties of *H. speciosa* (var. *speciosa* and var. *gardneri*).

*H. speciosa* plant material was collected in the state of Paraíba, Brazil (7°30’53”S; 34°53’07”W), and total DNA was extracted (including nuclear, chloroplast, and mitochondrial DNA) from approximately 2 cm^2^ of leaves following the cetyltrimethylammonium bromide extraction method, according to the protocol in Doyle and Doyle (1990) without modifications. The quantity and quality of the extracted DNA was verified by visualization on 1% agarose gel electrophoresis. The DNA samples were fragmented into 400–600 bp fragments using a mechanical procedure to construct the sequencing paired-end library. The fragments were ligated with adapters using “Nextera DNA Sample Preparation” (Illumina Inc., San Diego, CA, USA) according to the manufacturer's instructions, and 2×100 bp paired-ends were sequenced on the Illumina HiSeq2500 platform. Sequencing was performed at the Central Laboratory for High Performance Technologies in Life Sciences (LaCTAD-*Laboratório Central de Tecnologias de Alto Desempenho em Ciências da Vida*) at the State University of Campinas-UNICAMP, SP, Brazil.

The reads were trimmed using BBDuk and were used as input for comparative graph-based clustering with Repeat Explorer software (Novak *et al.*, 2010) and Tandem Repeat Analyzer (TAREAN) software (Novák *et al.*, 2017) implemented in the Galaxy environment (http://repeatexplorer-elixir.cerit-sc.cz) to identify satDNA, using repeatmasker database to provide information for annotation. The Repeat Explorer analysis allowed us to detect the genomic proportion of repetitive DNA, while TAREAN is a computational pipeline used to identify satDNAs from unassembled sequence reads. Paired-end reads of *A. syriaca*, *R. stricta*, and *C. roseus* were obtained from the NCBI and used for repeat identification and the comparative analysis in Apocynaceae.

The flow cytometry analysis revealed that the genome size ranged of the 2C-values 0.87 ± 0.02 and 0.88 ± 0.01 pg for *H. speciosa* var. *speciosa* and *H. speciosa* var*. gairdneri*, respectively, which corresponded to 1C-value of 430 Mb (1C values are measured in picograms, with 1 pg equivalent to 978 Mb). Pairwise comparisons among botanical varieties using Tukey's test (*p* < 0.05) showed that the botanical varieties had similar genomes (Table S1). Graph-based clustering revealed that the repetitive fraction corresponded to 38.24% in *H. speciosa*, 49.23% in *C. roseus*, 38.85% in *R. stricta*, and 74.18% in *A. syriaca* (Table S1). Clusters representing repeats making up at least 0.01% of the genome were characterized, of which the results for each species were 162 clusters in *H. speciosa,* 316 in *C. roseus,* 225 in *R. stricta*, and 239 in *A. syriaca,* corresponding to 19.87%, 36.63%, 23.025% and 51.674%, respectively (Figure 1 and Table 1). The repeatitive DNA showed that TEs were more abundant in all genomes, of which the LTR retrotransposons, including Ty1/*copia* and Ty3/*Gypsy* represented the major proportion. Abundance of the LTR retrotransposons ranged from 5.62% (*H. speciosa*) to 25.535% (*S. syriaca*), while the non-LTR was in a minor proportion or absent in some species (Table 1).

**Figure 1:**
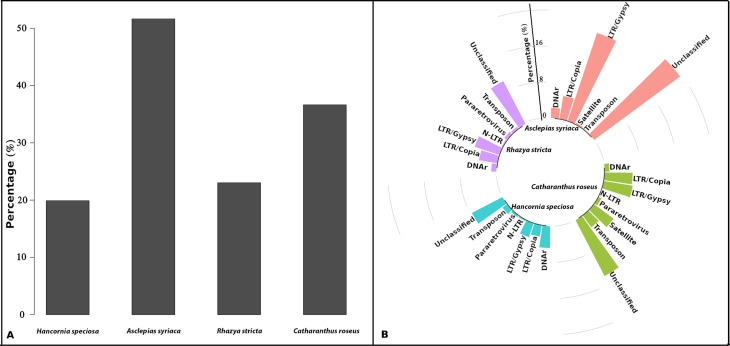
Graph-based clustering results. Repetitive DNA abundance (A) and characterization (B) of the repetitive DNA fraction in the *Hancornia speciosa*, *Asclepias syriaca*, *Rhazya stricta*, and *Catharanthus roseus* genomes.

**Table 1:** Genome proportions of repetitive sequences in *Hancornia speciosa*, *Catharanthus roseus, Rhazya stricta and Asclepias syriaca*.

Class	Order	Superfamily	Family	Genome [%]			
				*H. speciosa*	*C. roseus*	*R. stricta*	*A. syriaca*
**Retrotransposons**	LTR	Ty1-Copia	Ale	0.342	0.801	0.678	0.606
			Angela	0.250	0.850	0.355	0
			Bianca	0.042	0.015	0.273	2.078
			Ikeros	1.699	0.3	0	0.04
			Ivana/Oryco	0	0.0099	0.721	0.427
			SIRE	0	1.628	1.049	0
			TAR	0.1	0.571	0.18	0
			Tork	0.052	1.97	0.808	1.947
		**Total**		**2.485**	**6.1449**	**4.064**	**5.098**
		Ty3- Gypsy	Athila	0.3	1.302	1.702	15.58
			Chromovirus	2.662	5.103	2.941	2.18
			Ogre/Tat	0.171	0	1.066	2.677
		**Total**		**3.133**	**6.405**	**5.709**	**20.437**
		Retrovirus	Pararetrovirus	0.38	0.68	0.735	0
	Non-LTR (LINE)	L1		0.22	0.019	0.22	0
**Total**				**6.218**	**13.249**	**10.728**	**25.535**
**Transposons**	TIR	CACTA		0.459	1.179	0.05	0.021
		hAT		0.771	0.542	0.14	0
		PIF-Harbinger		0	0	0.026	0
		Mutator		0	1.439	0.265	0.042
		**Total**		**1.23**	**3.16**	**0.481**	**0.063**
	Helitron	Helitron		0.011	0	0	0
**Total (TEs)**				**7.459**	**16.409**	**11.209**	**25.598**
**rDNA**				4.818	1.008	1.134	2.22
**satDNAs**				0	5.332	0	0.274
**Total unidentified**				7.609	13.882	10.682	23.582
**Total Repeat**				**19.886**	**36.631**	**23.025**	**51.674**

Among the LTR Ty3/*Gypsy*, the chromovirus family was most abundant in *H. speciosa*, *C. roseus*, and *R. stricta,* which corresponded to 2.662%, 5.103%, and 2.941%, respectively, while the Athila family was more abundant in *A. syriaca* with 15.58% of the genome (Table 1 and Figure S1). A similar distribution among species was observed for the Ty1/*Copia* families, in which a major abundance of the Bianca family was detected in *A. syriaca* (Table 1 and Figure S1). Among the transposons, Mutator was more abundant in *C. roseus* (1.439%); however, some elements were absent in *H. speciosa*, *R. stricta*, and *A. syriaca* (Table 1).

The high-throughput search for satDNAs revealed eight satDNAs in *C. roseus* (corresponding to 5.332% of the genome) and seven satDNAs in *A. syriaca* (corresponding to 0.274% of the genome), whereas satDNAs were not found for *H. speciosa* or *R. stricta* (Table 1 and Figure S1). Moderately repetitive DNA was detected in the rDNA, ranging from 1% (*C. roseus*) to 4.818% (*H. speciosa*) (Table 1). Repeat DNA Ty3/*Gypsy* was interlaced on the rDNA intergenic spacer, resulting in the increase of the intergenic spacer (Figure S2).

Repetitive DNA sequences are characterized as highest repetitive in eukaryotic genomes, which increases genome sizes (Kazazian Jr, 2004). Among the components of the repetitive DNA, TEs are most important and correspond to the highest proportion of the genome (Feschotte *et al.*, 2008). The present study analyzed the fraction of repetitive DNA sequences in the three members of the Apocynaceae family and compared them to the fraction in the *H. speciosa* genome. We obtained *H. speciosa* (subfamily Rauvolfioideae) paired-end reads and paired-end reads of *R. stricta* (subfamily Rauvolfioideae), *C. roseus* (subfamily Rauvolfioideae), and *A. syriaca* (subfamily Asclepiodeae) were obtained from NCBI. Genome size is an important estimate of the amount of DNA in the cell nucleus and is known as the C-value (Pellicer *et al.*, 2018). The genome size estimate is classified as a small genome when the 1C-value is < 3.5 pg (Kelly and Leitch, 2011). Accordingly, we concluded that *H. speciosa* has a small genome and there was some minor variation across botanical varieties. The small genome size of *H. speciosa* (average 1C-value = 0.44 pg), together with other Apocynaceae species (Table S1 and Plant DNA C-values Database - https://cvalues.science.kew.org/), suggests that the family may be characterized with a small genome size. Genome size may be positively correlated with the repetitive fraction; however, the *A. syriaca* genome was highly repetitive, with a genome size 1C-value of 0.42 pg. The high proportion of repetitive DNA detected in *A. syriaca* may be due to the high abundance of the LTR retrotransposon (25.535%), of which 15.58% corresponded to the Athila family, while the repetitive DNA in other species may be compounded by several families with minor proportions that were undetectable by graph-based clustering.

Characterization of the *H. speciosa* genome revealed a greater abundance of the LTR retrotransposon, of which the chromovirus had a greater proportion. This characteristic is similar to other species of Apocynaceae, in which the LTR retrotransposons were more abundant in all of the genomes analyzed. SatDNA was absent in *H. speciosa* and *R. stricta*, suggesting that transposable elements may have replaced the typical satDNA. LTR retrotransposons are localized in the centromeres of plants (Weber and Schmidt, 2009) and the LTRs *Gypsy*/Chromovirus have a domain associated with chromatin, which interacts with centromeric proteins (Nagaki *et al.*, 2003). Future molecular cytogenetics studies may clarify whether chromovirus replaced the satDNA in *H. speciosa.* We conclude that *H. speciosa* has a small genome characterized predominantly by LTR retrotransposons and the absence of satDNA.[Bibr B26]

## Supplementary material

The following online material is available for this article.

Figure S1 - Characterization of transposable elements and satDNA in the genomes of *Asclepias syriaca* (A), *Catharanthus roseus* (B), *Hancornia speciosa* (C), and *Rhazya stricta* (D).

Figure S2 - Graph-based clustering of *Hancornia speciosa* rDNA.

Table S1 - Quantification of genome size and repetitive DNA analysis in *Hancornia speciosa*, *Catharanthus roseus*, *Rhazya stricta*, and *Asclepias syriaca*.
