# Supplementary material for: Genome size and identification of repetitive DNA sequences using low coverage sequencing in Hancornia speciosa Gomes (Apocynaceae: Gentianales)
Source: Genet Mol Biol. 2020 Nov 9;43(4):e20190175. doi: 10.1590/1678-4685-GMB-2019-0175 (PMC7654370; doi:10.1590/1678-4685-GMB-2019-0175)
Supplement: Supplementary file 1 [file 1415-4757-GMB-43-4-e20190175-s3.pdf]

Genetics and Molecular Biology
https://doi.org/10.1590/1678-4685-GMB-2019-0175

Supplementary Material to "Genome size and identification of repetitive DNA sequences using low coverage sequencing in *Hancornia speciosa* Gomes (Apocynaceae: Gentianales)"

Table S1 - Quantification of genome size and repetitive DNA analysis in *Hancornia speciosa*, *Catharanthus roseus, Rhazya stricta and Asclepias syriaca.*

Species	Genome size [pg - Mb]	Analyzed reads	Coverage	Repetitive fraction (%)	References[b]
H. speciosa	0.44 - 430	660744	0.07	38.24	This study
C. roseus	0.76 – 743	661788	0.08	49.23	Guimarães et al. (2012)
R. stricta	0.28 – 274[a]	660754	0.24	38.85	Sabir et al. (2016)
A. syriaca	0.42 – 411	661946	0.16	74.18	Bai et al. (2012)

a – estimated; b- references for genome size estimate
