# Supplementary material for: Genome size and identification of repetitive DNA sequences using low coverage sequencing in Hancornia speciosa Gomes (Apocynaceae: Gentianales)
Source: Genet Mol Biol. 2020 Nov 9;43(4):e20190175. doi: 10.1590/1678-4685-GMB-2019-0175 (PMC7654370; doi:10.1590/1678-4685-GMB-2019-0175)

*Genetics and Molecular Biology*
https://doi.org/10.1590/1678-4685-GMB-2019-0175

# Supplementary Material to "Genome size and identification of repetitive DNA sequences using low coverage sequencing in *Hancornia speciosa* Gomes (Apocynaceae: Gentianales)"

**Figure S1 -** Characterization of transposable elements and satDNA in the genomes of *Asclepias syriaca* (A), *Catharanthus roseus* (B), *Hancornia speciosa* (C), and *Rhazya stricta* (D).

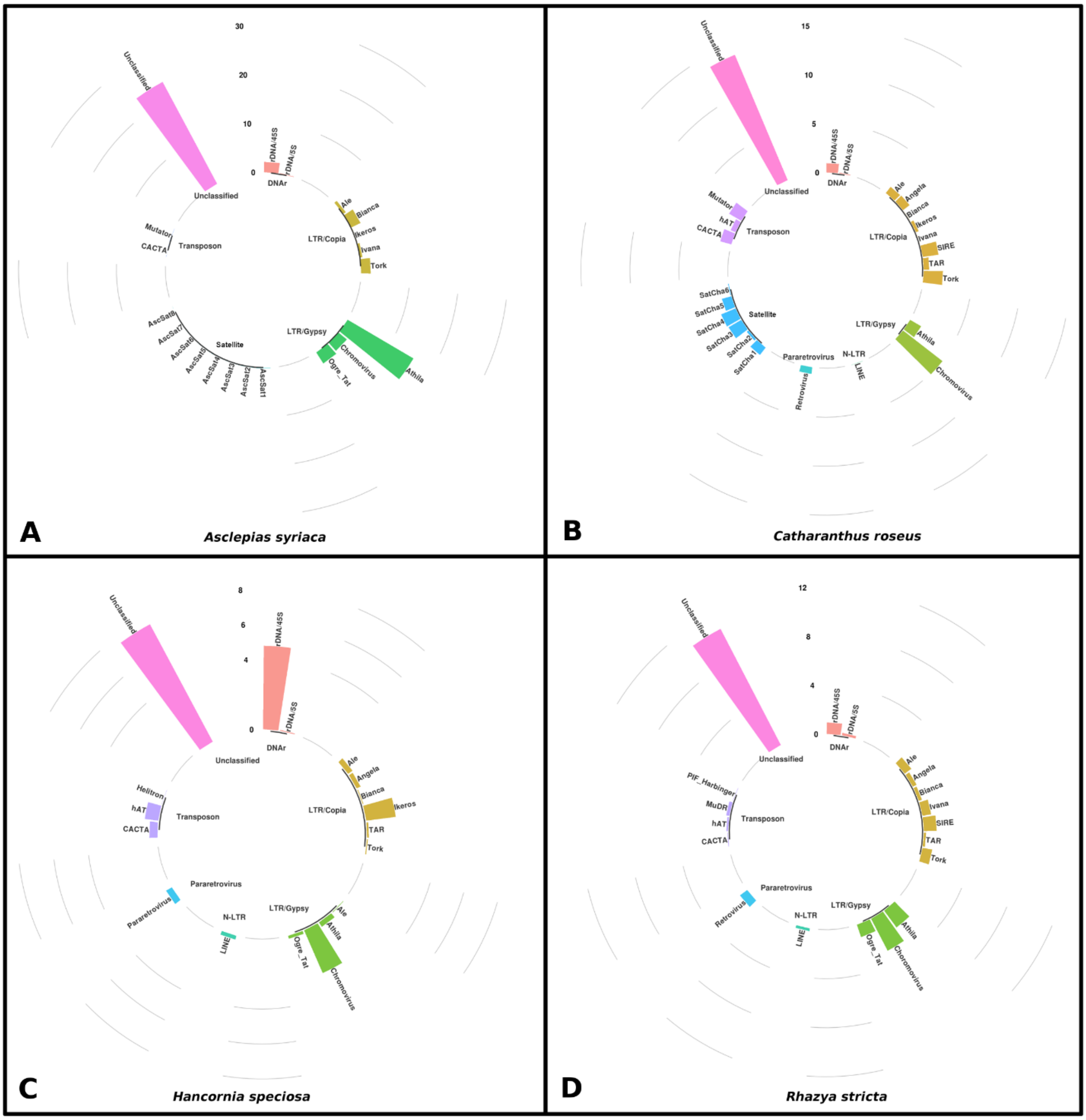

Supplement: Supplementary file 2 [file 1415-4757-GMB-43-4-e20190175-s1.pdf]
