# Supplementary material for: Genome size and identification of repetitive DNA sequences using low coverage sequencing in Hancornia speciosa Gomes (Apocynaceae: Gentianales)
Source: Genet Mol Biol. 2020 Nov 9;43(4):e20190175. doi: 10.1590/1678-4685-GMB-2019-0175 (PMC7654370; doi:10.1590/1678-4685-GMB-2019-0175)

Genetics and Molecular Biology
https://doi.org/10.1590/1678-4685-GMB-2019-0175

Supplementary Material to "Genome size and identification of repetitive DNA sequences using low coverage sequencing in *Hancornia speciosa* Gomes (Apocynaceae: Gentianales)"

Figure S2 - Graph-based clustering of *Hancornia speciosa* rDNA. The circle represents the clusters and the colors represent the rDNA regions.

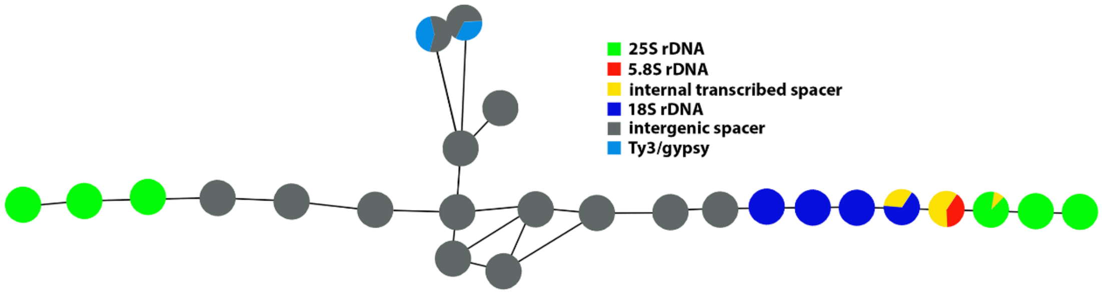

Supplement: Supplementary file 3 [file 1415-4757-GMB-43-4-e20190175-s2.pdf]
